# 

**Published:** 1834-04-01

**Authors:** 


### MEDICAL

### QUARTERLY REVIEW.

N.Y. ACADEMY LIBRARY OF MEDICINE.

VOL. II.

1453

LONDON:

J. SOUTER, 73, ST. PAUL'S CHURCH-YARD;

SOLD ALSO BY BURGESS AND HILL; J. CHURCHILL; E. COX; J. HIGHLEY; RENSHAW AND RUSH, &c.

W. JACKSON, NEW YORK, AGENT FOR THE UNITED STATES.

1834.

J. AND C. ADLARD, PRINTERS,
BARTHOLOMEW CLOSE.

### NOTICES.

*We have received* Dr. PROUT'S *Bridgewater Treatise,* Dr. MARSHALL HALL *on Diagnosis,* Dr. BOW *on Inflammation,* Mr. KIERNAN *on the Liver,* Mr. DALRYMPLE *on the Eye, a Pamphlet on Medical Education and Reform, by a Licentiate of the College of Physicians, and* Dr. RAMADGE *on Consumption. We shall review them in our next.*

*We have also received a new work by* Mr. PERCIVAL, *entitled "Hippopathology." It does not fall within our plan to review it, but we shall give an extract or two from it in the Collectanea of our next Number.*

Mr. VALENTINE, *and* Mr. WM. TAYLOR, *will find the substance of their Communications in our Collectanea.*

*We must request our Contributors to favour us with their Communications before the* 16*th of the month preceding publication.*

*"Literary Intelligence," or, in other words, advertisements of books about to be published, can appear only in our Advertising Sheet.*

